# Microstructure, Fatigue Properties and Stress Concentration Analysis of 6005 Aluminum Alloy MIG Welded Lap Joint

**DOI:** 10.3390/ma15217729

**Published:** 2022-11-02

**Authors:** Yanlei Li, Shanglei Yang, Zeng Peng, Zhentao Wang, Zihao Gao

**Affiliations:** 1 School of Materials Engineering, Shanghai University of Engineering Science, Shanghai 201620, China; 2 Shanghai Laser Intelligent Manufacturing and Quality Inspection Professional Technical Service Platform, Shanghai 201620, China

**Keywords:** MIG welding, 6005 aluminum alloy, fatigue, stress concentration, lap joint

## Abstract

This paper studies the microstructure and mechanical properties of MIG (Melt Inert Gas) lap welded 6005 aluminum alloy plates. Microstructure analysis (OM) of the joint showed that 15~30 μm small grains were observed at the fusion line. Mechanical analysis shows that the small grains are broken by shielding gas and molten pool flow force. Hardness test shows that there is a softening zone (41~43 HV) in HAZ much lower than BM and WZ. The low cycle fatigue test showed that the performance of lap joint decreased sharply, and the fatigue strength of weld decreased significantly, which was only 27.34% of the base metal. The fatigue fracture (SEM) of the weld observed slip band cracking and a large number of brittle fracture characteristics. Using the stress concentration factor $K_{t}$ for analysis, it was found that the cause of brittle fracture was mostly stress concentration. Lap joint stress concentration model appears in two ways: firstly, at the weld toe, the weld is subjected to eccentric force, secondly, there is a small gap between the two plates at the weld root, which cracks along the direction of 45° of the maximum shear stress.

## 1. Introduction

With the rapid development of aerospace and transportation, the demand for lightweight metals is increasing. Traditional steel is gradually being replaced by metal materials with high strength-to-weight ratio because its large weight cannot meet the requirements of some structures. Aluminum alloy is one of the most common materials to reduce the weight of structures. It has excellent properties, such as high strength, high stiffness, high recycling value, fast heat conduction, easy molding, and light weight [1,2,3]. 6005 aluminum alloy is a common Al-Mg-Si alloy, which is mainly composed of Al matrix and precipitated strengthening phase [4]. It is widely used in transportation vehicles, marine vessels, high-speed trains, and other manufacturing industries. [1,4].

In the manufacturing process, the structure is sometimes too large to be cut and cast in one whole. In this case, it is necessary to use joining technology to connect the small parts together. Welding is the most typical joining technology. MIG (Melt Inert Gas) welding is the most widely welding method for 6005 aluminum alloy, which is widely used in automatic welding production lines [5,6,7,8]. However, during MIG welding of aluminum alloys, the thermal properties and chemical composition of aluminum alloys are easy to cause defects such as deformation, softening, and increased porosity. These defects also cause some subsequent effects, such as: deformation can cause post-weld cracks, residual stress; softening can cause strength reduction and uneven mechanical properties; the increase of porosity can cause the decrease of surface quality and weld quality. In addition, deformation can cause defects such as post-weld cracks and residual stress. Softening can cause strength reduction and uneven mechanical properties. The increase of porosity can cause the decrease of surface quality and weld quality. These conditions will lead to a decline in the mechanical properties of aluminum alloys, and poor organizational composition of aluminum alloys, resulting in reduced availability of welding metals [8,9,10,11,12,13], and a serious reduction in mechanical properties, which causes the greatest harm to society. Therefore, in order to avoid major hazards to society, it is necessary to pre-evaluate the mechanical properties of welded joints. Fatigue testing is a common way to evaluate mechanical properties [9,10,14,15]. It is characterized by reflecting the durability and fracture characteristics of materials under long-term service. The main purpose of fatigue testing has three aspects: measuring the fatigue life of materials, studying the fatigue fracture process, and studying the influencing factors of fatigue life. Several researchers have studied the effect of MIG welding on the fatigue life of aluminum alloy, and shown that low surface temperature, porosity, wear, and corrosion are all unfavorable to the fatigue life of aluminum alloy [9,10,15,16]. When fatigue testing is performed, the load type, stress level, load ratio, joint morphology, and stress concentration of welded joints are several important factors that aggravate fatigue crack initiation and propagation [17,18,19]. In the fatigue test of T-joints and lap joints, the fatigue performance often decreases, mainly due to large stress concentration caused by uneven joint morphology [20,21,22,23,24]. With the increase of stress concentration, the time of crack initiation is shortened, and crack initiation increases the risk of sudden fracture [25]. There is a significant correlation between joint morphology and stress concentrations, which can be larger within lap joints and can sometimes be a major cause of fatigue fracture, adding extra risk of use [20,21,22]. Therefore, it is of great significance to study the fatigue fracture and its inducement of welded lap joints [24].

In order to provide some references for the safety of 6005 aluminum alloy and the welding MIG method in practical use. This paper uses MIG welding 6005 aluminum alloy lap joint to study the welding quality, microstructure, hardness, fatigue and stress concentration of 6005 aluminum alloy lap joint under this method.

## 2. Test Materials and Methods

### 2.1. Materials

The base metal is 6005 aluminum alloy plate with a thickness of 6 mm. Welding wire is ER5365 alloy with a diameter of 1.3 mm. The proportion of component content (weight percentage, %) of base metal and welding wire is shown in Table 1.

### 2.2. Methods

**Figure 1:**
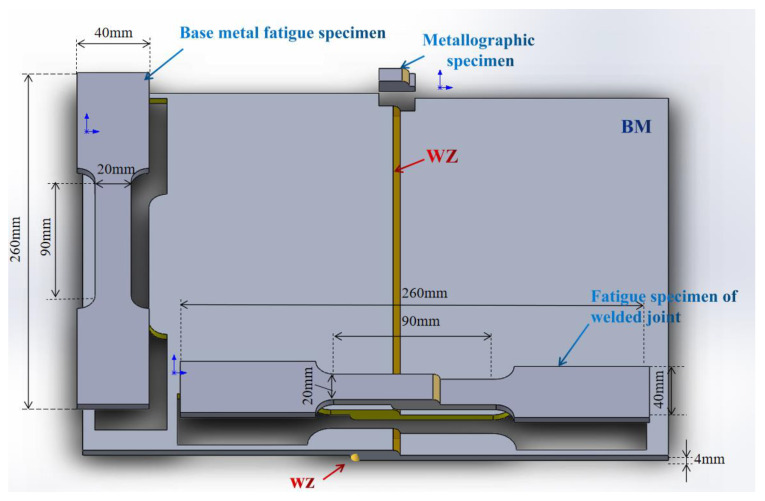
Fatigue and Metallographic (hardness) Samples.

The test material was lap welded using MIG welding method. Welding parameters: current 150 A, voltage 20 V, welding speed 6 mm/s. The shielding gas flow rate was 20 L/min. Argon (concentration 99.99%) was used to protect the welding process. Before welding, the two plates are overlapped with an overlap length of 20 mm. MIG welding is performed on both sides of the overlap. After welding, the base metal fatigue sample, weld fatigue sample, and metallographic sample (hardness sample) were cut by wire cutting, and the size is shown in Figure 1.

The hardness of the welded samples was tested using HDX-1000 Vickers hardness tester of Shanghai Taiming Optical Instrument Co., Ltd., Shanghai, China with a loading load of 0.98 N and a holding time of 15 S. The value interval was selected every 0.2 mm, with a full length of about 13 mm.

The metallographic samples were grinded with 600#, 800#, 1000#, 1200# and 1500# sandpaper in turn until smooth and without scratches. Polished weld joints were etched with Keller reagent (2 mL HF + 3 mL HCl + 5 mL HNO3) to corrode the alumina layer and the grain boundary [26]. The etched weld joints were metallographically observed under the 4XC optical microscope (OM) of Shanghai Taiming Optical Instrument Co., Ltd., Shanghai, China.

Before the fatigue test, the fatigue samples were grinded with polishing machine. According to the national standard GB/T15248-2008 (axial constant amplitude low cycle fatigue test method for metallic materials), the HB250 electro-hydraulic servo tester of Zwick/Roell Amsler, Germany is used to perform low cycle fatigue test on base materials and welded joints at room temperature. The frequency is 15 Hz, the stress ratio is *R* =$\;\sigma_{max}$*/*$\sigma_{min}\;$= 0.1, and the loading method is tension- tension fatigue test. The fatigue fracture was cleaned by an ultrasonic cleaner to remove the alumina layer and surface impurities, and then observed under S-3400 N scanning electron microscope (SEM) of Hitachi, Tokyo, Japan.

## 3. Results 

### 3.1. Microstructure Analysis of 6005 Aluminium Alloy Lap Joint

**Figure 2:**
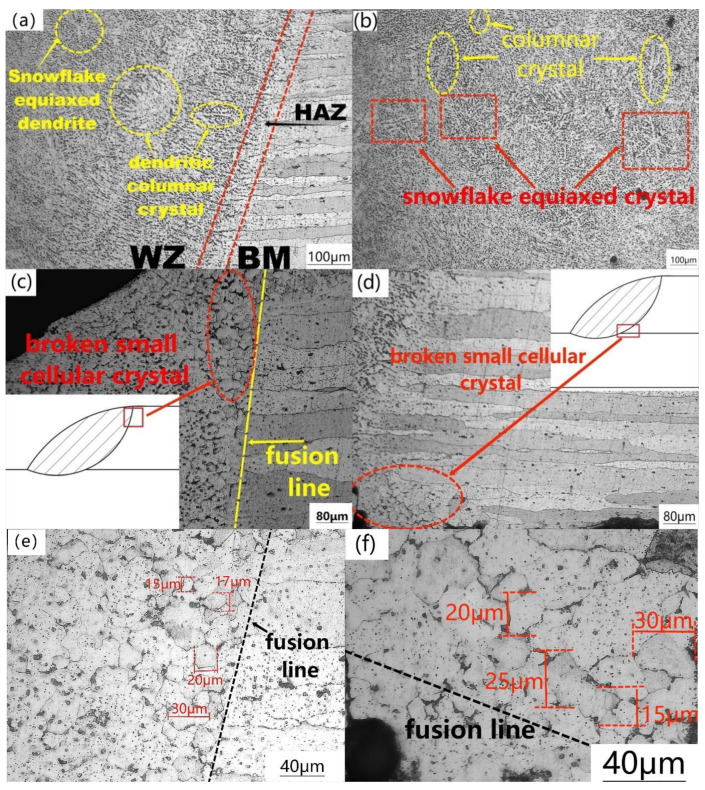
Microstructure of 6005 aluminum alloy MIG welded lap joint: (**a**) fusion line, (**b**) weld center, (**c**) broken grains in the upper right corner of the fusion line, (**d**) broken grains in the lower right corner of the fusion line, (**e**) enlarged view of the upper right corner, (**f**) enlarged view of the lower right corner.

The welding boundary of 6005 aluminum alloy lap joint (OM) is shown in Figure 2a. Within the heat affected zone (HAZ) can be observed the microstructure altered by the welding heat. In the weld zone (WZ), the transformation of solidification microstructure from columnar crystal to equiaxed crystal can be seen. Figure 2b shows the weld center, and there are a large number of coarse columnar crystals and snowflake equiaxed crystals, which are caused by the growth time and undercooling. When the weld is solidified, the undercooling of the weld boundary is small, resulting in the same grain orientation, pointing to the cooling gradient direction to generate columnar crystals. However, the undercooling of the weld center is large, the grain orientation increases, and the growth time is long, forming snowflake equiaxed grains [27].

Figure 2c,d show the small broken grains near the fusion line of the welded joint where the specific positions are drawn in the white box, and the broken grains only appear in these two places. This is due to the interruption of columnar crystals during growth by arc, shielding gas, surface tension, buoyancy, etc. [27,28,29]. These small grains prove that MIG welding has a certain effect on grain refinement. Figure 2e,f is an enlarged view of these two places. The sizes of these small grains were measured to be about 15–30 μm.

### 3.2. Molten Pool Force Model Analysis of Broken Grains

**Figure 3:**
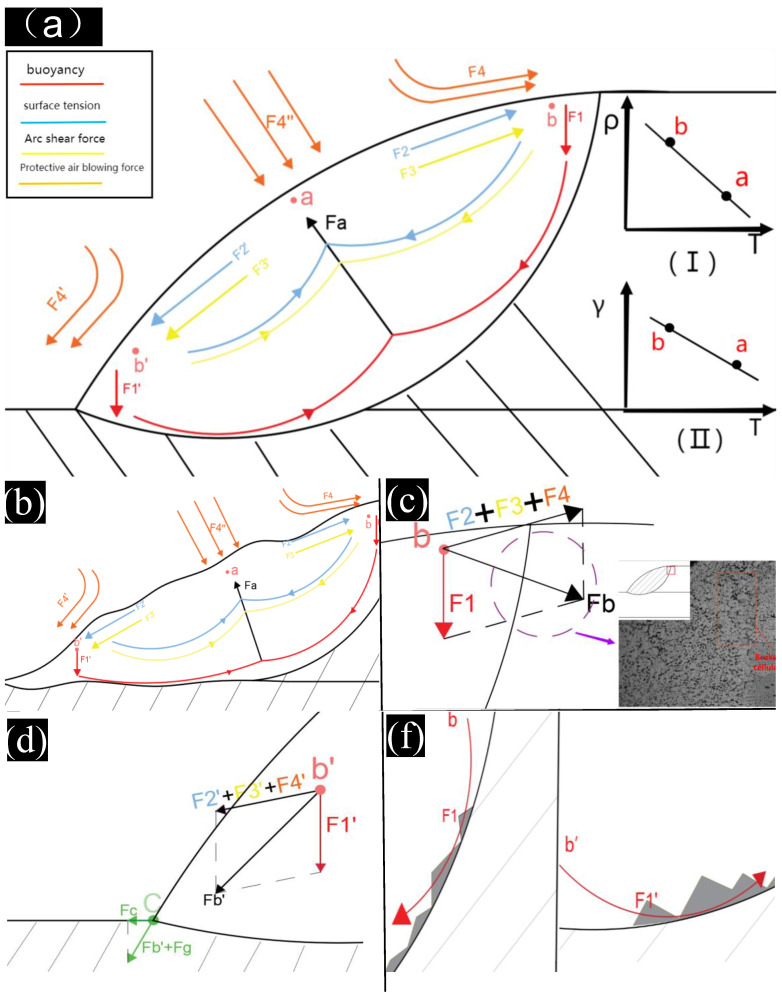

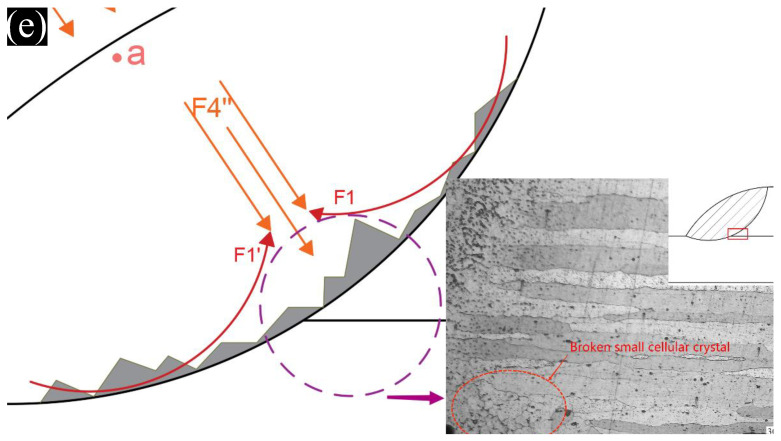
Stress analysis of the broken grains: (**a**) weld pool of the lap joint, (**b**) collapse morphology, (**c**) upper right corner of the fusion line, (**d**) lower left corner of the fusion line, (**e**) weld bottom of the fusion line, (**f**) other positions of the fusion line.

Figure 3a shows the force diagram of the welding pool of a lap joint. The model reference is the dynamic description of molten pool flow by Kou, S [27]. Four forces are described in this model: metal buoyancy, surface tension, arc shear stress and protective air blowing force. At the beginning of solidification, the relationship between density and temperature of the points with higher temperature (point *a* in the center of the molten pool) and lower temperature (points *b* and *b′* near the fusion line) is shown in Figure 3a(I). In low temperature places, some liquid metals solidify first, which increases the density. The first solidified liquid metals sink under the influence of gravity, forming a downward force *F1* near the fusion line, and the vacancy generated by the downward metal flow will be supplemented by the liquid metal on the left side of *b*, and the vacancy on the left side needs to be supplemented by the liquid in the middle, so a force pointing from the bottom of the weld to the center of the weld is formed, which is incorporated into *Fa*. The relationship between surface tension *γ* and temperature *T* is shown in Figure 3a(II). In the absence of surfactant, the surface tension decreases with the increase of temperature [30]. Point *a* has a higher temperature and a lower surface tension, while point *b* and *b′* have a higher surface tension and a lower temperature. The point with a higher surface tension will pull the liquid metal towards this point, forming a force *F2* pointing to the fusion zone. The force formed by the flow of liquid merges into *Fa*. The plasma moves outward at high speed along the surface of the molten pool and forms an outward shear stress *F3* on the surface of the molten pool. The liquid metal is carried by force and returned under the molten pool and incorporated into resultant force *Fa*. *F4* is the protective gas blowing force for welding, the maximum blowing force is vertical to the center of the weld, while encountering the surface of the molten pool away from the center of the weld will change direction slightly, as shown in Figure 3a.

The causes of the broken grains in Figure 2c,d are shown in Figure 3c, referring to the force analysis at point *b*, which is subjected to arc shear force, surface tension and protective air blowing force. These three forces are generally parallel in directions and are therefore used as a force (*F2 + F3 + F4*). This force is combined with the buoyancy *F1* at point *b* to form a resultant force *Fb* pointing to the fusion line.

Figure 3e shows the force diagram at the bottom of the molten pool. The protective air blow is the largest of the four forces that occur, of which the *F4*′′ of the vertical weld is the largest. At the bottom of the weld, the return force of surface tension and arc shear cannot reach this position. The only forces against *F4* are buoyancy return forces *F1* and *F1*′. The return force of buoyancy is less than protective gas blowing force. When liquid metal solidifies and accumulates at the bottom, the return force of buoyancy consumes energy by bypassing the accumulated metal, reducing *F1* and *F1′*, which cannot prevent *F4*″ from directly impacting the bottom of the weld. The broken grains at the bottom of the weld are caused by direct impact of protective gas.

Figure 3d shows a stress analysis of *b*′. Force *F2*′ *+ F3*′ *+ F4*′ is along the weld and force *F1*′ is vertically down form force *Fb*′. Direction points to the toe of the weld. However, two points need to be noted: the right side of point *b* is unmelted base material, which cannot cause excessive deformation. Most of the energy is used to promote flow cycle and impact the base material. If this energy is not consumed after solidification, residual stress will also be formed. Moreover, unlike *b*, there is no rigid object on the left side of *b*′, which can deform. Most of the energy is converted into kinetic energy, causing the molten pool to collapse forward. Then the energy used to impact the fusion line is reduced.

Figure 3b shows the morphology after the collapse. Taking point *c* of the weld toe as an example, there is also an impact force *Fg* of molten pool liquid flowing down the surface under the influence of gravity. The force *Fg* is approximately regarded as the same direction as *Fb*′, and the resultant force *Fb*′ *+ Fg* points obliquely downward to the base metal, because the base metal is a rigid object, and the force will be converted into a force *Fc* along the surface of the base metal to the left. So, most of the energy at *b*″ will eventually be converted into the deformations driven by *Fc*. This is the reason why there is no grain breakage at the lower left corner of the fusion line.

No broken grains appear from point *b* down and point *b*′ to the right. This is because these locations are too deep and only buoyant flow forces *F1* and *F1*′ are present, which are small and parallel to the fusion line, and do not directly impact the fusion line. The solidified metal deposited at the bottom will also block the liquid flow and reduce these forces, as shown in Figure 3f.

### 3.3. Hardness Analysis

**Figure 4:**
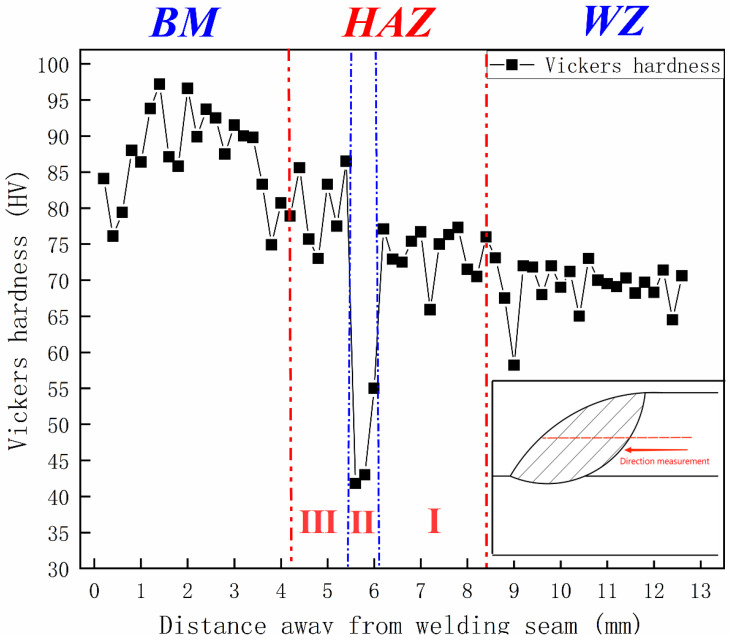
Weld hardness.

Figure 4 shows the hardness indentation profile of 6005 aluminum alloy lap joint as indicated by the inset. The measurement direction is shown in the lower right box. The average hardness of BM, HAZ and WZ is 88 HV, 72 HV and 69 HV respectively, and the average hardness of WZ and HAZ is 78% and 82% of BM. The lowest hardness is located in zone II of HAZ, about 41~43 HV.

Mxz et al. studied the hardness of 6xxx aluminum alloy [4]: the hardening of 6xxx aluminum alloy is mainly solution-hardening. The solute is soluble in Al and forms lattice distortion, which can hinder the movement of dislocations to achieve the hardening of aluminum alloy. The solution-strengthening phase of 6005 aluminum alloy is mainly β phase (${\mathrm{Mg}}_{2}$Si) and its intermediate phase $\beta^{\prime }$ and $\beta^{\prime \prime }$. Among them, the strengthening effect of phase $\beta^{\prime \prime }$ is the strongest and phase $\beta^{\prime }$ is the weakest. The precipitation temperatures of these strengthening phases are respectively $\beta^{\prime \prime }$ 160~240 °C, $\beta^{\prime }$ 240~380 °C, β 450~550 °C. Therefore, with the increase of the distance from the weld, precipitation under the influence of welding thermal cycle corresponds respectively to β, $\beta^{\prime }$, $\beta^{\prime \prime }\;$ [4,27,31]. Kou, S [27] mentioned that the decrease in hardness of WZ and HAZ of 6xxx aluminum is due to the large loss of phases $\beta^{\prime \prime }$ and the coarseness of the microstructure after heating.

HAZ is affected by the welding thermal cycle, and the farther away from the heat source, the lower the temperature. Therefore, the main strengthening phases of zones I, II and III in the HAZ are respectively to β, $\beta^{\prime }$, $\beta^{\prime \prime }$. The strengthening phase produced in Zone III of HAZ is mainly the $\beta^{\prime \prime }$ phase with precipitation temperature of 160~240 °C, but by the influence of heat source, there will still be some phase re-dissolution and transformation, making the hardness of zone III slightly lower than that of BM. Zone II is the softening zone of HAZ, and this zone is the area where $\beta^{\prime }$ is abundantly precipitated. There are three reasons for the severe decrease in hardness. First, $\beta^{\prime }$ is a phase that does not contribute significantly to the hardness. Secondly, the temperature is higher than that of zone III, resulting in coarser grains. Finally, the factors that make the hardness decrease in zone III are also active in zone II, which also subject to re-dissolution and transformation; Zone I is the side close to the weld, which is subject to high temperature and reaches the the precipitation temperature of β phase. The factors that reduce the hardness of zone II and III will also occur in zone I, and there will also be the generation and growth of $\beta^{\prime }$ and $\beta^{\prime \prime }$, but the main factors in this zone are β Phase, which contributes more than $\beta^{\prime }$ to the hardness, so that the hardness of this zone is reduced, but it will not fall to the degree of softening zone II.

WZ due to complete melting, strengthening phase loss is more serious. The greater the heat, the coarser the grains, and the lower the hardness.

### 3.4. Fatigue Analysis

According to the national standard GB/T 24176-2009 (fatigue test data statistics and analysis method), 11 groups of fatigue data of base metal and welded joints are selected. The function of stress amplitude $S_{a\;}\;$= ($\sigma_{max}$ − $\sigma_{min}$)/2 and fatigue life *N* is selected for research, and the relationship between the two is Formula (1)

(1) $$S_{a}^{k}\cdot N=C$$

where *k* is the fatigue strength coefficient and *C* is the material constant. For the convenience of research, the exponential relation of Formula (1) is transformed into logarithmic relation (2), and its linear relation can be seen.

(2) $$logN=-k\cdot logS_{a}+logC$$

**Figure 5:**
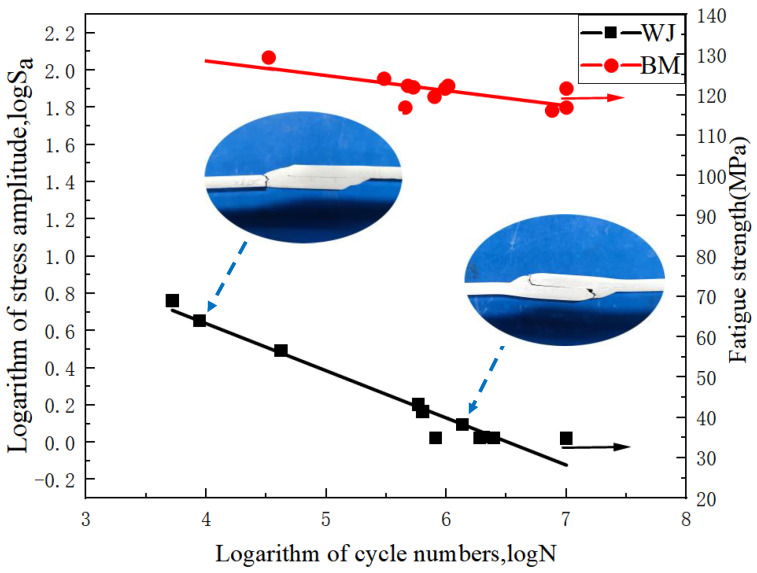
S-N fatigue curve of base metal and welded joint.

According to Formula (2), the fatigue data is fitted by the origin software, and the parameters of the curve are fitted and calculated. Then the fatigue S-N curve of the welded joint and the base metal is drawn, Figure 5. The parameters of test result of 6005 base metal and welded joint are shown in Formulas (3) and (4).

(3) $$logN=-0.08096\cdot logS_{a}+log168.78$$

(4) $$logN=-0.23317\cdot logS_{a}+log58.90$$

The comparison between the two formulas shows that the *k* value of the welded joint is relatively large, which is 2.88 times higher than that of the base metal. The *C* value is small, and is 2.86 times lower than the base metal. *k* is negatively correlated with fatigue performance, and the greater the value of *k*, the more severe the decline of fatigue performance. *C* is also meaningful under certain conditions. When *N* = 1, *C* is negatively correlated with *k* and positively correlated with fatigue performance. Because it is under the action of one cycle, *C* can also represent the tensile capacity of materials to a certain extent, but it is not accurate and can only be used for reference. The fatigue strengths of the welded joint and base metal are 32.75 Mpa and 119.78 Mpa respectively, and the fatigue strength of weld decreases significantly, being only 27.34% of that of the base metal.

Images and calculations show that with the increase of $S_{a}$, the fatigue life *N* decreases, and the fatigue life of lap joint decreases faster than that of base metal. At higher stress levels, the weld samples more likely to fracture at the toe, and at low stress levels at the bottom of the weld. These two places are the stress concentration points of the lap joint.

### 3.5. Fatigue Fracture Analysis

**Figure 6:**
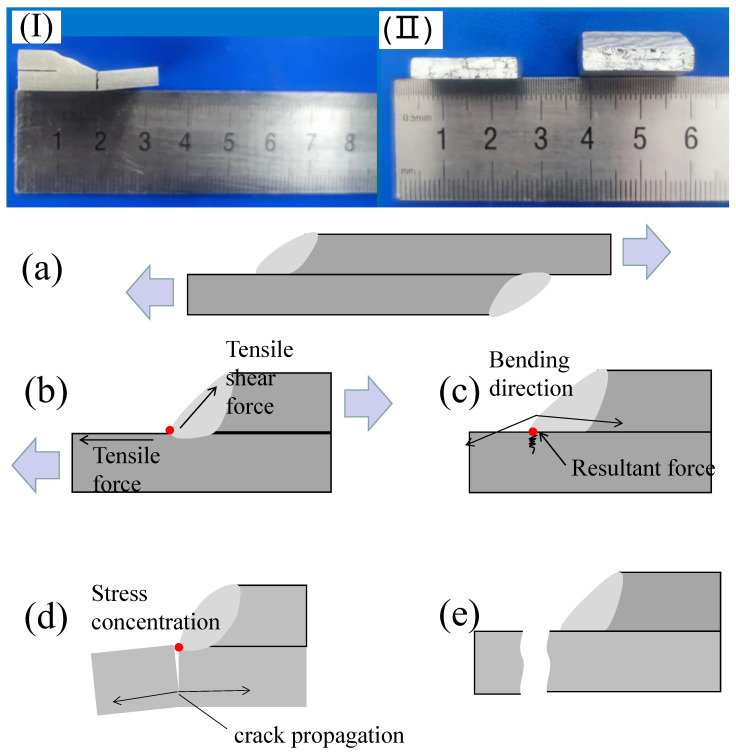
Stress concentration fracture mechanism of lap joint: (**I**) fracture location, (**II**) fracture morphology, (**a**) fatigue specimen and loading direction, (**b**) stress distribution at weld toe, (**c**) stress concentration and sample bending, (**d**) crack propagation, (**e**) specimen fracture.

Figure 6I,II shows that the fatigue fracture position of the 6005 aluminum alloy lap joint is located at the weld toe, extending through to the bottom plate below. The lower bottom plate was bent during the fracture process because of the stress concentration at the weld toe of the lap weld. During the fatigue test, the sample is subjected to two directions of loading and the toe joint is approximately understood to be subjected to a tensile shear stress along the weld surface and a positive tension of the plate, as shown in Figure 6a,b. The two forces synthesize a resultant force pointing to the weld toe, which concentrates the stress at the weld toe. Under the effects of this force, plastic bending of the fatigue sample occurs, producing microcracks, as shown in Figure 6c. When the stress concentration reaches the fatigue crack threshold value $\Delta K_{\mathrm{th}}$, the crack begins to expand until it breaks, as shown in Figure 6d,e. This lap welding structure itself has stress concentration, and in the microscopic case, without plastic fracture, large brittle fractures occur, reducing the weld performance. 

**Figure 7:**
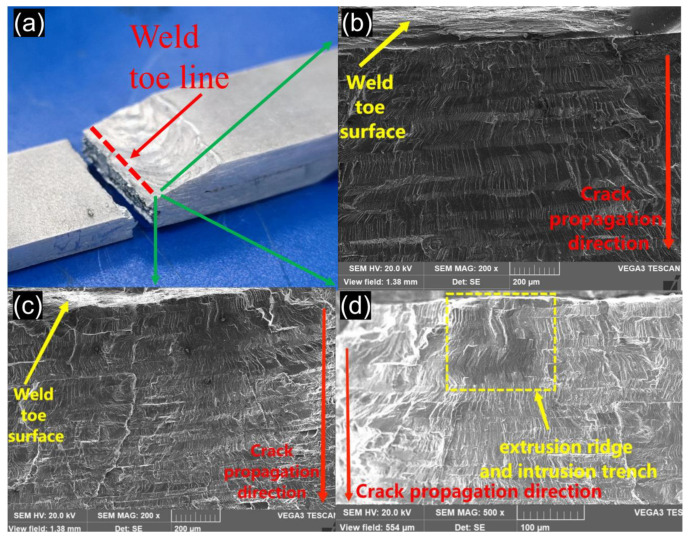
SEM image of weld toe line cracking: (**a**) weld toe line, (**b**,**c**) River pattern, (**d**) small fatigue source.

Because of the high stress concentration of the lap joint, each point on the weld toe line is conducive to fracture, which ultimately leads to cracking of the weld sample from the entire weld toe line and a brittle fracture over a large area, as seen in Figure 7a. Therefore, no obvious fatigue source zone can be observed on the fracture surface, and on the contrary, there are a large number of brittle fracture characteristics. Figure 7b,c shows two brittle fractures (SEM) that start at the toe line of the weld with a distinct riverlike pattern that extends all around the object. Figure 7d is a small source of fatigue, which is caused by slip band cracking. The obvious features of the slip band cracking can be seen: extrusion ridges (bright color) and intrusion trenches (dark color). Because the notch in the source region repeatedly opens and closes during the crack propagation process, resulting in large friction, the fracture is generally bright. The location of fatigue source region and the mode of initiation of crack can be further inferred to be the crack of slip band caused by stress concentration.

**Figure 8:**
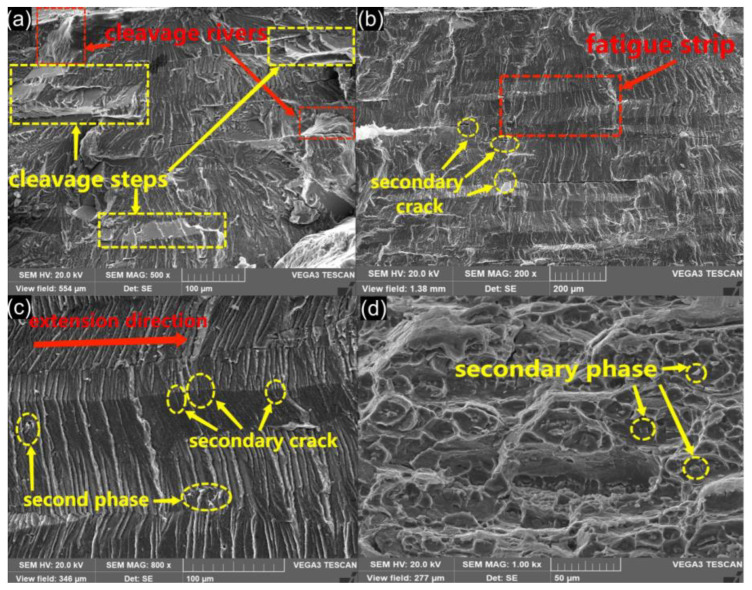
SEM image: (**a**) cleavage river and cleavage step, (**b**) fatigue crack growth zone, (**c**) fatigue bands, (**d**) base metal dimple.

There are many obvious brittle fracture morphologies in the fatigue crack growth zone. Figure 8a is cleavage fracture (SEM), the red box is cleavage river, and the yellow box is cleavage step. Both are typical features of cleavage fracture. Cleavage fracture is a kind of low-energy fracture phenomenon that occurs along some low-index crystallographic planes of metals or alloys under normal stress, which is mostly brittle. Figure 8b is a SEM image of fatigue crack growth area of 6005 aluminum alloy lap joint. The expansion area shows beach line pattern macroscopically, and the expansion direction is from up to down. The secondary crack in the yellow ring is perpendicular to the direction of the main crack growth, which is the mode of intergranular growth. The crack propagation along the intergranular path consumes less energy and is characterized by brittleness. Fatigue bands can be seen in the red box. A fatigue band can be regarded as one or more stress cycles. Figure 8c is an enlarged photo of the fatigue band in red box of Figure 8b. The fatigue bands are relatively neat, and can thus be considered plastic fatigue bands. Fatigue bands are not completely parallel because grain boundaries, impurities, and the secondary phase can hinder crack growth during fatigue crack growth, resulting in asynchronous fatigue band growth. The lower yellow circles show second phases of about 3~4 μm in size, which differ in appearance from the fatigue bands and hinder fatigue bands progress. The upper yellow circles show secondary cracks, which are often accompanied by fatigue bands in the fatigue crack growth, parallel to the direction of fatigue bands. The specimens are fractured in this manner in the direction of crack propagation until fracture.

No dimples were found in the lap joint fracture which proves that 6005 aluminum alloy is ductile-brittle, but dimples were found in the fatigue fracture of the base metal, as shown in Figure 8d. Within the yellow rings is a broken second phase, about 2~4 μm in size. The broken second phase is at the bottom of the dimple because it blocks dislocation movement and increases toughness during dimple enlargement. The second phase is torn up in this repeated dislocation motion. The dimples found in the base metal fracture indicate that 6005 aluminum alloy is a toughness material. However, welding and lap morphology can degrade the material properties and result in brittle fracture. There is no stress concentration on the morphology of the base metal, resulting in a plastic fracture.

## 4. Discussion

### 4.1. Stress Concentration Factor of 6005A Lap Joint

The stress concentration factor $K_{t}$ is used to study the stress concentration of the lap joint. The expression is shown in Formula (5):

(5) $$K_{t}=\sigma_{max}/\sigma_{n}=169\;\mathrm{MPa}/149\;\mathrm{MPa}=1.13$$

where $\sigma_{max}$ is the maximum stress and $\sigma_{n}$ is the nominal stress at fracture, used the preferred data from the base metal tensile test. The stress concentration factor $K_{t}$ is used to evaluate the notch sensitivity of the specimen. If $K_{t}$ > 1, the material is not sensitive to notch, if $K_{t}$ < 1, the fracture stress is less than the maximum tensile strength and there is no obvious necking phenomenon. The engineering stress-engineering strain curve shows a low plastic curve, and the smaller $K_{t}$ is, the closer the curve is to the brittle material curve, and the fracture is brittle fracture.

**Figure 9:**
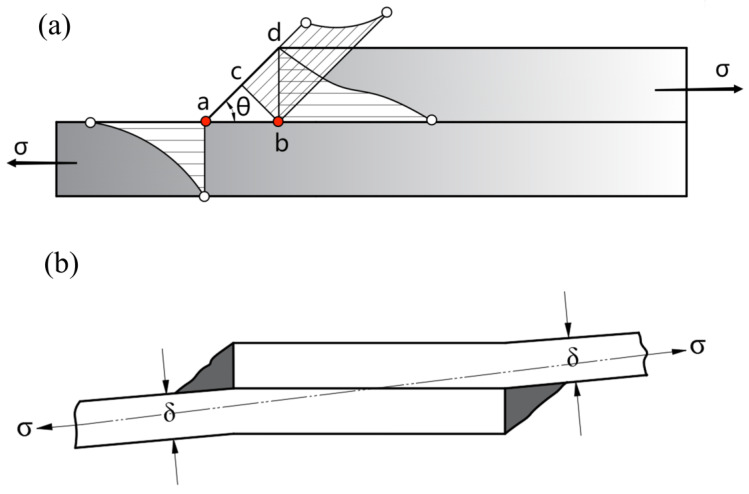
Stress distribution of 6005 lap welded joint during loading: (**a**) stress distribution field, (**b**) bending morphology at point *a* stress concentration.

The stress distribution at the stress concentration point is shown in point *a* of Figure 9a. The greater the inclination θ of the weld, the greater the stress gradient and the greater the stress concentration factor. The stress concentration point *a* is in the state of two- direction tensile force. The two forces are axial stress and tangential stress. According to Hooke’s theorem, Formula (6) can be obtained:

(6) $$\{\begin{array}{c} \sigma_{1}=K_{t}\sigma_{n}\\ \;\sigma_{t}=\nu K_{t}\sigma_{n} \end{array}\;$$

where $\sigma_{1}$ is axial stress, $\sigma_{t}$ is tangential stress and ν is Poisson’s ratio. The stresses in two directions are combined into a force pointing to the weld toe as shown in Figure 6c, Formula (7). Substituting Formula (6) into it gives:

(7) $$\sigma^{*}={\left[\sigma_{1}^{2}+\sigma_{t}^{2}+{\left(\sigma_{1}-\sigma_{t}\right)}^{2}\right]}^{1/2}/\sqrt{2}=K_{t}\sigma_{n}(1-\nu +\nu^{2}{)}^{1/2}\;$$

where $\sigma^{*}$ is the composite stress of two-direction stress. The combined stress is significantly greater than the axial stress in tension, so to calculate the stress concentration factor at point *a*, the maximum stress $\sigma_{max}$ of the lap joint should be replaced by $\sigma^{*}$ that of the joint. This fives the Formula (8):

(8) $$K_{t}^{\prime }=\sigma^{*}/\sigma_{n}=K_{t}(1-\nu +\nu^{2}{)}^{1/2}=1.13\text{×}\sqrt{\left(1-0.345+0.345^{2}\right)}=0.994$$

where $K_{t}^{\prime }$ is the composite stress concentration factor under two-direction tension, and the Poisson ratio of aluminum alloy is usually ν = 0.345. In conjunction with the foregoing, $K_{t}^{\prime }$ = 0.994 < 1, it can be concluded that the stress concentration at the toe of the lap welding increases the stress, increases the sensitivity of the sample to notches, and increases the probability of brittle fracture.

### 4.2. Stress Concentration Point Analysis of Overlap Welds

The stress distribution of the lap joint in the fatigue test is shown in Figure 9a. During the loading process, the stress distribution is not uniform, of which point *a* at the toe and point *b* at the root are the largest. The stress diagram at point *a* along the direction of *cd* shows that the shear stress at the weld surface increases with the increase of angle θ. The ideal mathematical model is Formula (9):

(9) $$\{\begin{array}{c} F^{\prime }=F/cos\theta ,\;(0<\theta <\frac{\pi }{2})\\ dF^{\prime }=F/cos\theta d\theta =F\cdot sec\theta tan\theta =F\cdot \frac{sin\theta }{cos\theta^{2}}>0,\;(0<\theta <\frac{\pi }{2}) \end{array}$$

where $F^{\prime }$ is the shear force in the cd direction and F is the axial force, $dF^{\prime }$ proves that the larger the angle, the greater the shear force. The left side of point *a* is the stress distribution diagram under the action of stress concentration. The closer to point *a*, the more obvious the stress concentration is. Due to the eccentricity between the overlap weld and the applied force, additional bending stress occurs in the joint under tension and the specimen is bent, as shown in Figure 9b. The greater the stress level, the greater the shear stress in the direction of *cd*, and the greater the resultant force. This is the reason why fatigue specimens often fracture at the weld toe at high stress levels.

**Figure 10:**
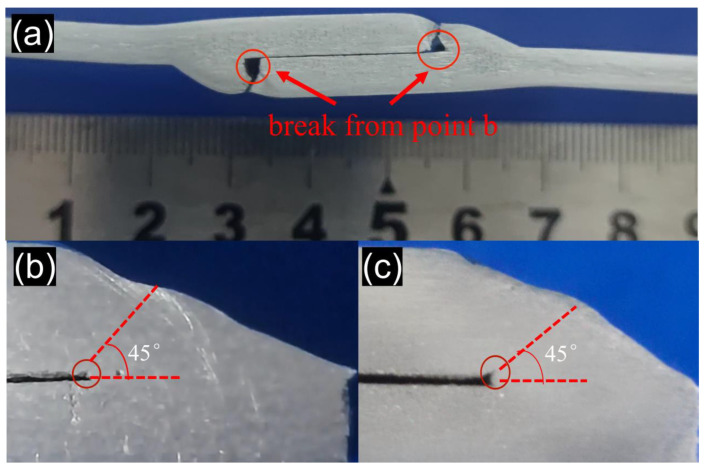

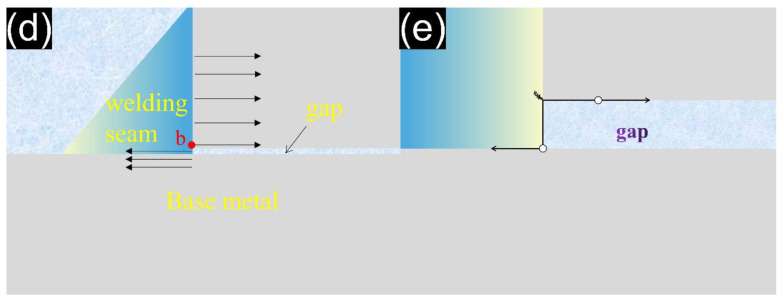
Stress concentration at weld bottom: (**a**) point *b* fracture, (**b**,**c**) 45 ° damage at point *b* when point *a* fracture, (**d**,**e**) stress concentration mechanism at point *b*.

The root position point *b* is also the stress concentration point of the lap joint, and the stress distribution diagram is on the right side of point *b*, Figure 9a. Figure 10a shows the fracture sample at the point *b* of weld root. The reason for the stress concentration is that the lap welding process between two plates cannot achieve close fitting, when the welding wire and base metal melted into one, but the non-melting zone cannot be completely fused, so there is a small gap in the plate area between the two welds, as shown in Figure 10d. Point *b* is an angle of 90° and the two-way tension is not on the same plane, where the stress must be concentrated. Cracks occur in the material under the influence of tensile stress as shown in Figure 10e, mostly at 45° in the direction of maximum shear stress due to morphological reasons. There are many factors influencing the stress concentration at point *b*. The stress concentration is sometimes lower than point *a*, and breaks at point *a*; and it is sometimes higher than point *a,* and breaks at point *b*. However, as a stress concentration point of lap joint, damage at point *b* can be observed even at point *a* break in the fatigue tests. Figure 10b,c shows the damage at point *b* when point *a* breaks and the direction of all cracks points to 45°.

## 5. Conclusions

In this study, two 6 mm thick 6005 aluminum alloy plates were lap welded by MIG welding. The characteristics consist of microstructure, mechanical properties, fracture analysis and stress concentration model. The relationship between stress concentration model and fatigue phenomenon was studied by observing the results, data, and photos (OM, SEM). The main conclusions are summarized as follows:

(1) The microstructure (OM) analysis shows that there are 15~30 μm grains broken by the flow force and shielding gas near the fusion line, which improves the weld quality. The force model of the molten pool found that the force is the largest at the weld toe and weld bottom on both sides, and broken grains appear at both the right weld toe and weld bottom. Since the left weld toe is not rigidly fixed, most of the energy is converted into kinetic energy, and no broken grains appear.
(2) Mechanical properties tests include hardness and fatigue. The lowest hardness is located in the softening zone of HAZ, about 41~43 HV. The softening zone is located on the side of the heat affected zone, which is different from the fatigue fracture position. Softening has nothing to do with fatigue fracture. Fatigue testing shows that the fatigue life of lap joint decreases fast. The fatigue strength of the weld and the base metal are 32.75 Mpa and 119.78 Mpa respectively, and the weld is only 27.34% of the base metal. Fatigue specimens tend to fracture at the point of stress concentration. At higher stress levels, weld specimens are mostly broken at the weld toe, and at low stress levels at the bottom of the weld.
(3) The fatigue fracture (SEM) shows that the welded specimen cracks from the whole weld toe line, and a large area of brittle fracture occurs without obvious fatigue source area. Brittle fracture characteristics such as cleavage river, cleavage step and secondary crack of intergranular propagation widely exist in crack propagation zone. The base metal sample has no stress concentration caused by the structure, resulting in plastic fracture. A large number of dimples appeared in the base metal fracture.
(4) A stress concentration model at weld root and weld toe was proposed. Stress concentration at the weld toe was found due to the eccentricity between the overlap weld and the applied force. Additional bending stress occurs in the joint under tension and the specimen is bent. At the weld root, because the two plates cannot be closely connected during the lap welding process, forming a 90° sharp angle, so the stress must be concentrated, and the crack probability is 45° in the direction of the maximum shear stress. We used the stress concentration factor $K_{t}$ to verify the stress concentration degree of the 6005 aluminum alloy lap joint. The results show that the stress concentration was related to the inclination of the weld, and it is proved to be sensitive to the notch in the case of 6005 aluminum alloy by MIG lap welding.

## Figures and Tables

**Table 1:** Chemical composition of 6005 aluminum alloy and ER5356 welding wire wt.%.

Material	Al	Mg	Zn	Cu	Cr	Si	Fe	Mn	Ti
6005	surplus	0.40~0.70	≤0.20	≤0.30	≤0.30	0.50~0.90	≤0.35	≤0.50	≤0.10
ER5365	surplus	≤0.05	≤0.10	≤0.30	0	4.50~6.00	≤0.80	≤0.05	≤0.20

## Data Availability

The data that support the findings of this study are available from the corresponding author upon reasonable request.
